# Evaluation of the Effects of *Cornus mas* L. Fruit Extract on Glycemic Control and Insulin Level in Type 2 Diabetic Adult Patients: A Randomized Double-Blind Placebo-Controlled Clinical Trial

**DOI:** 10.1155/2015/740954

**Published:** 2015-10-05

**Authors:** Rasool Soltani, Abdollah Gorji, Sedigheh Asgary, Nizal Sarrafzadegan, Mansour Siavash

**Affiliations:** ^1^Department of Clinical Pharmacy and Pharmacy Practice, Faculty of Pharmacy and Pharmaceutical Sciences, Isfahan University of Medical Sciences, Isfahan, Iran; ^2^Isfahan Cardiovascular Research Center, Isfahan Cardiovascular Research Institute, Isfahan University of Medical Sciences, Isfahan, Iran; ^3^Isfahan Endocrine & Metabolism Research Center, Isfahan University of Medical Sciences, Isfahan, Iran

## Abstract

*Background*. The plant *Cornus mas* L. (cornelian cherry) is traditionally used as an antidiabetic supplement; however, there is no related clinical trial. In this study, we evaluated the effects of the fruit extract of this plant on biomarkers of glycemic control in adult patients with type 2 diabetes. *Methods*. Sixty patients with type 2 diabetes were randomly assigned to two groups to receive either the extract or placebo capsules (2 capsules twice daily) for 6 weeks. Each drug capsule contained 150 mg of anthocyanins. Fasting plasma levels of glucose, insulin, HgbA_1C_, and triglyceride as well as 2-hour postprandial glucose level (2Hpp) were measured before and after the intervention and finally the mean values were compared between groups. *Results*. After 6 weeks of intervention, significant increase in insulin level (1.13 ± 1.90 versus −0.643 ± 1.82, *P* < 0.05) as well as decrease in HgbA_1C_ (−0.24 ± 0.429 versus 0.023 ± 0.225, *P* < 0.05) and TG (−23.66 ± 55.40 versus 2.83 ± 15.71, *P* < 0.05) levels was observed in drug group compared to placebo. *Conclusion*. Daily consumption of the fruit extract of *Cornus mas* L. improves glycemic control by increasing insulin level and reduces TG serum level in type 2 diabetic adult patients.

## 1. Introduction

Diabetes mellitus (DM) is one of the most common endocrine disorders characterized by hyperglycemia. Type 2 DM, characterized by insulin resistance and a relative lack of insulin secretion, accounts for as much as 90% of all cases of DM and its prevalence is increasing [1]. DM is the leading cause of blindness in adults aged 20 to 74 years and end-stage renal disease (ESRD) and a main cause of cardiovascular events [1]. Optimal management of the patient with DM will reduce or prevent complications and improve quality of life [2]. Also, aggressive management of cardiovascular risk factors, including dyslipidemia, is needed to reduce the likelihood of development of macrovascular disease [2].

Medical nutrition therapy is recommended for all patients with DM and, along with activity, is a cornerstone of treatment [3].* Cornus mas* L. (cornelian cherry) is a plant found in parts of central and southern Europe as well as western Asia including northern forests of Iran [4]. The fruits (berries) of this plant are rich in anthocyanins including delphinidin-3-glucoside, cyanidin-3-rhamnoglucoside, cyanidin-3-glucoside, cyanidin-3-galactoside, and pelargonidin-3-galactoside [4, 5]. It has been shown that anthocyanins increase insulin secretion from pancreatic *β*-cells and improve insulin resistance [6–9]. Furthermore, a recent animal study has shown the effect of* Cornus mas* L. fruit on reduction of blood glucose level in diabetic rats [10]. Although this plant is traditionally used as an antidiabetic supplement, there is no clinical study about its effect. Therefore, this trial aimed to evaluate the effects of* Cornus mas* L. fruit extract on several markers of glycemic control in type 2 diabetic adult patients.

## 2. Materials and Methods

### 2.1. Plant Material and Extraction

Fresh ripe berries of* C. mas* were collected from the forests of Ghazvin, Iran, in July 2012. After washing and separation of the cores, the fruits were crushed by electric mixer (Moulinex, France) and filtrated by filter paper. The obtained material was then extracted by maceration with ethanol 70% (Stalk, Iran) repeated for 3 times. The extract was then filtrated and concentrated using rotary evaporator (Heidolph, Germany).

### 2.2. Extract Standardization

The obtained extract was standardized based on the total anthocyanin content using the pH differential method [11]. For this, two 1-g samples of dried extract were dissolved in 10 mL of buffer solution with pH = 1 composed of 125 mL of KCl 0.2 M (Merck, Germany) and 375 mL of HCl 0.2 M (Merck, Germany) and 10 mL of buffer solution with pH = 4.5 composed of 400 mL of sodium acetate 1 M (Merck, Germany), 240 mL of HCl 1 M, and 360 mL of water, respectively. Both solutions were diluted 10 times with the same buffer and their absorbance was read at 510 nm using spectrophotometer (PerkinElmer, USA). Total anthocyanin content was determined by the following equation:

(1) Anthocyanin  concentration  (mg/L) =(AbspH 1−AbspH 4.5)×484.82×1000×DF24825,

where 484.82 is the molecular mass of cyanidin-3-glucoside chloride, 24825 is molar absorptivity of cyanidin-3-glucoside at 510 nm in pH = 1, and DF is the dilution factor.

### 2.3. Preparation of Drug and Placebo Capsules

The concentrated extract was mixed with tribasic calcium phosphate powder (Merck, Germany), then granulated, and dried. Each drug capsule was filled with 500 mg of the mixed granules equivalent to 150 mg of total anthocyanin. The placebo capsules with shape, color, and size similar to drug ones were filled only with dried granulated tribasic calcium phosphate.

### 2.4. Patient Selection

The inclusion criteria for participation of patients in the study were (1) being diagnosed with type 2 diabetes mellitus for at least 2 years according to the American Diabetes Association (ADA) diagnostic criteria [12], (2) age of 18 to 80 years, (3) serum glycosylated hemoglobin (HbA_1C_) > 7% and <10%, (4) not being substance abuser (including alcohol), (5) not using any insulin preparation and/or any antidiabetic drug increasing endogenous insulin secretion (sulfonylureas, glinides, glucagon-like peptide-1 (GLP-1) analogs, and dipeptidyl peptidase IV (DPP-IV) inhibitors), (6) no change of the dose of antidiabetic drug within the last month, (7) free of either liver, kidney, or cardiovascular disease, (8) free of diabetic foot ulcer, and (9) not being pregnant or lactating (for women).

The exclusion criteria included (1) irregular use of the capsules, (2) change of the dose of antidiabetic drug during the study, and (3) the need to use any insulin preparation and/or any antidiabetic drug increasing endogenous insulin secretion (as mentioned above).

### 2.5. Study Design and Interventions

This was a randomized, double-blind, placebo-controlled clinical trial conducted in Isfahan Cardiovascular Research Center affiliated to Isfahan University of Medical Sciences, Isfahan, Iran, from December 2012 to September 2013. Informed consent was obtained from all participants and the study protocol was approved by the Ethical Committee of Isfahan University of Medical Sciences. Patients who met the inclusion criteria were randomly and equally assigned to either the study drug (*Cornus mas*) or placebo groups. Before intervention, the demographic characteristics (including BMI) were recorded for all patients and, by receiving 5 mL of blood sample from each participant in fasting state and 2 hours after meal, fasting serum levels of glucose (FPG), insulin, and triglyceride (TG), glycosylated hemoglobin (HbA_1C_), and serum level of 2-hour postprandial glucose (2Hpp) were determined. Also, to detect any possible side effect of the drug on the liver and kidney, the serum levels of alanine aminotransferase (ALT), aspartate aminotransferase (AST), blood urea nitrogen (BUN), and creatinine were obtained. The patients of drug and placebo groups were instructed to use 4 medicinal and placebo capsules, respectively, per day (2 capsules every 12 hours) with food for 6 weeks. All patients were advised to maintain their usual diet and physical activity and report any adverse effect during the study. The patients' compliance was evaluated by counting their capsules at the end of use and their results were applied for data analysis if they used more than 80% of their capsules. At the end of 6 weeks, all the above-mentioned variables were again determined and compared to baseline values. For randomization and blindness, each capsule container was given a code according to the type of its content (drug or placebo). When giving a container to each patient, its code was recorded on his/her own consent form. At the end of the intervention and after determination of the patient's own results, the recorded code was identified in terms of the type of intervention. All participants, the physician, and the laboratory personnel were blind to the intervention type.

### 2.6. Statistical Analysis

SPSS 20.0 software (SPSS Inc., Chicago, USA) was used for statistical analysis of obtained data. Kolmogorov-Smirnov test was performed to assess distribution pattern of continuous data. Because of normal distribution of all continuous data, Student's *t*-test was used for comparisons. Paired-samples *t*-test was performed for comparison of values at the beginning and end of intervention within each group. Independent-samples *t*-test was used for comparing the mean changes of each parameter form baseline between drug and placebo groups. Chi-square test was done for comparison of gender distribution in two groups. *P* < 0.05 was considered as significant.

## 3. Results

**Figure 1:**
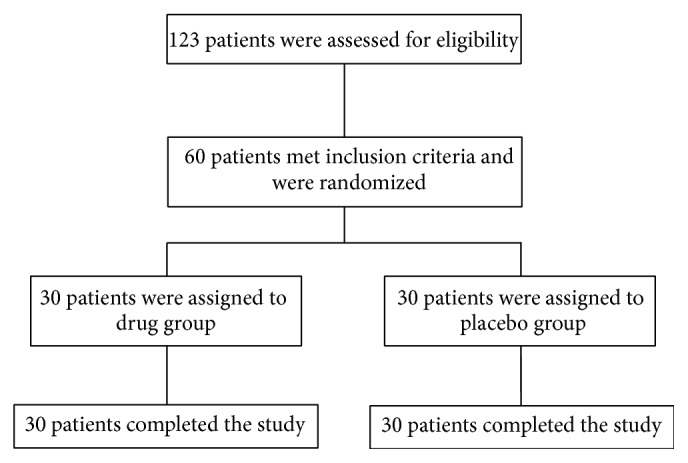
Flowchart of patients' enrollment in the study.

During the study, a total of 123 type 2 diabetic patients were assessed for participation in the study, of whom 60 patients (age range of 41 to 65 years) met the inclusion criteria that were randomly and equally divided into two intervention groups (30 in each group). All patients fully completed the trial (Figure 1).

Table 1 shows baseline demographic and clinical characteristics of the study patients. As shown, all subjects were matched regarding baseline values.

Table 2 shows comparatively the effects of interventions on evaluated variables after 6 weeks in the study patients. As seen,* C. mas* significantly reduced the serum levels of HbA_1C_ and TG and increased the serum level of insulin compared to placebo. Although* C. mas* reduced BMI, FPG, and 2Hpp, these effects were not statistically significant compared to placebo.

Table 3 presents the effects of* C. mas* and placebo on laboratory markers of liver and kidney function after 6 weeks of intervention. As shown, no significant changes were detected in these values during the study. Also, no complication or adverse effect was reported by the patients of both groups.

## 4. Discussion

Our study showed that the fruit extract of* C. mas* could improve glycemic control characterized by reduction of HbA_1C_ as measurements of HbA_1C_ are the gold standard for following long-term glycemic control [13]. Although the effects of this plant on FPG and 2Hpp were not statistically significant compared to placebo, the significant decrease in FPG compared to baseline (*P* < 0.05) and slight decrease in 2Hpp show the potential ability of this extract for reduction of serum glucose level. Therefore, the use of higher doses of the extract for longer periods of time might have more significant effect on markers of glycemic control in type 2 diabetic patients. The observed effects of* C. mas* fruits may be due to its anthocyanin content. It has been shown that the anthocyanins cyanidin-3-glucoside and delphinidin-3-glucoside, found in the fruits, stimulate insulin secretion from rodent pancreatic beta-cells (INS-1 832/13) in vitro [7]. This is consistent with our results showing increased level of insulin by the extract. Also, the study of Zhang et al. showed that the compound ursolic acid in fruits of* C. mas* is capable of phosphorylation of insulin receptors and stimulation of glucose uptake by tissues [14]. In an animal study performed by Jayaprakasam et al. the anthocyanins and ursolic acid, purified from* C. mas* fruits, prevented glucose intolerance and increased insulin levels in high-fat-fed mice [6]. Furthermore, release of acetylcholine to raise insulin secretion through stimulation of muscarinic type 3 (M3) receptors by oleanolic acid contained in* Cornus* species, as suggested by Hsu et al. [15], may be another mechanism of increased insulin level and consequent reduction of glucose level.

To the best of our knowledge, there is no clinical trial about the effects of cornelian cherry on glycemic control in diabetic patients. However, several animal studies have been conducted on* Cornus mas* [6, 10, 16] and* Cornus officinalis* [17, 18]. In the study of Shamsi et al., daily use of 2 g of* Cornus mas* fruits by alloxan-induced diabetic rats for 4 weeks resulted in significant reduction of blood glucose levels, increased insulin levels, and increased size of pancreatic islets compared to control nondiabetic rats [10]. In the study of Yamabe et al., treatment with* Cornus officinalis* fruit extract for 10 days suppressed hyperglycemia, proteinuria, and renal advanced glycation end-product (AGE) formation in streptozocin-induced diabetic rats [18].

Since a common lipid abnormality in type 2 diabetes is hypertriglyceridemia (>150 mg/dL) [19] as was present in our study subjects, we evaluated the effect of* Cornus mas* on triglyceride levels too in the study subjects. The extract showed significant TG-lowering effect in type 2 diabetic patients. This effect could be due to anthocyanin content of the extract as the hypolipidemic effects of anthocyanins and some anthocyanin-containing plants have been confirmed in several studies [20–23]. The TG-lowering effect of anthocyanins may be through suppression of the expression of lipogenic enzymes (fatty acid synthase, acyl-CoA synthase 1, and glycerol-3-phosphate acyltransferase) in the liver and adipose tissue [24] as well as increasing lipoprotein lipase activity in skeletal muscle and reducing it in visceral adipose tissue [25].

It is noteworthy that the large standard deviation (SD) of change values from baseline (as shown in Table 2) could be due to several factors including relatively low number of patients in each group and large variations in the change of each parameter in several patients, both presenting some limitations of the current study.

In conclusion, our study shows that daily consumption of the fruit extract of* Cornus mas* L. (equivalent to 600 mg of anthocyanins daily) improves glycemic control by increasing insulin level and reduces TG serum level in type 2 diabetic adult patients. Therefore, this extract could be considered as a beneficial nutritional supplement for adult patients with type 2 diabetes. However, more studies with larger sample size and longer duration are required to confirm these results.

## Figures and Tables

**Table 1:** Baseline demographic and clinical characteristics of the study subjects. The values are presented as mean ± SD.

Parameter (unit)	*Cornus mas *	Placebo	*P* value
	(*n* = 30)	(*n* = 30)	
Age (years)	49.16 ± 5.62	49.93 ± 6.12	0.616
Gender (%male)	63.33	66.70	0.723
BMI (kg/m^2^)	29.40 ± 1.73	29.21 ± 2.01	0.621
FPG (mg/dL)	157.93 ± 41.38	174.67 ± 40.80	0.120
Insulin (*μ*U/mL)	5.67 ± 2.85	5.91 ± 2.72	0.727
HbA_1C_ (%)	7.72 ± 0.75	7.78 ± 0.65	0.745
2Hpp (mg/dL)	228.40 ± 66.42	231.20 ± 60.27	0.865
TG (mg/dL)	198.37 ± 66.64	237.43 ± 148.95	0.615

BMI: body mass index; FPG: fasting plasma glucose; 2Hpp: 2-hour postprandial glucose; TG: triglyceride.

**Table 2:** The effects of interventions on tested parameters after 6 weeks in the study subjects. The values are presented as mean ± SD.

Parameter	*Cornus mas *	Placebo	*P* value
(unit)	(*n* = 30)	(*n* = 30)	(between groups)
BMI (kg/m^2^)			
End	29.06 ± 1.60	29.31 ± 2.07	0.062
Change	−0.33 ± 0.45	0.10 ± 0.45	
*P* value	0.723	0.320	
FPG (mg/dL)			
End	143.30 ± 40.19	178.73 ± 38.80	0.130
Change	−14.63 ± 36.87	4.06 ± 55.39	
*P* value	0.038	0.691	
Insulin (*μ*U/mL)			
End	6.80 ± 3.20	5.27 ± 2.53	0.001
Change	1.13 ± 1.90	−0.643 ± 1.82	
*P* value	0.003	0.064	
HbA_1C_ (%)			
End	7.49 ± 0.71	7.81 ± 0.64	0.005
Change	−0.240 ± 0.429	0.023 ± 0.225	
*P* value	0.005	0.621	
2Hpp (mg/dL)			
End	222.17 ± 55.17	244.83 ± 63.27	0.247
Change	−6.23 ± 46.19	13.63 ± 80.76	
*P* value	0.466	0.363	
TG (mg/dL)			
End	174.70 ± 78.28	240.26 ± 147.97	0.014
Change	−23.66 ± 55.40	2.83 ± 15.71	
*P* value	0.026	0.332	

BMI: body mass index; FPG: fasting plasma glucose; 2Hpp: 2-hour postprandial glucose; TG: triglyceride.

**(a):** 

Parameter (unit)	*Cornus mas* (*n* = 30)		
	Baseline	Week 6	*P* value
ALT (U/L)	16.43 ± 9.61	16.70 ± 6.47	0.848
AST (U/L)	22.76 ± 6.75	23.36 ± 7.07	0.673
BUN (mg/dL)	16.01 ± 2.77	15.88 ± 3.65	0.879
Creatinine (mg/dL)	0.633 ± 0.225	0.593 ± 0.316	0.335

**(b):** 

Parameter (unit)	Placebo (*n* = 30)		
	Baseline	Week 6	*P* value
ALT (U/L)	17.46 ± 8.16	17.86 ± 7.44	0.830
AST (U/L)	22.16 ± 9.83	23.76 ± 14.13	0.447
BUN (mg/dL)	15.41 ± 2.73	15.73 ± 3.49	0.677
Creatinine (mg/dL)	0.620 ± 0.274	0.713 ± 0.278	0.232
